# Beyond expensive innovations: affordable and effective strategies for managing tubulopathies in adults

**DOI:** 10.1093/ckj/sfag001

**Published:** 2026-01-09

**Authors:** Louise Nielsen, Lucile Figueres

**Affiliations:** Nephrology, Association ECHO, Nantes, France; Nantes Université, CHU Nantes, INSERM, Center for Research in Transplantation and Translational Immunology, UMR 1064, ITUN, Nantes, France; Reference Centers for Rare Diseases of Calcium and Phosphate (Filière OSCAR) and Nephrogenetic (Filière ORKID), Nantes, France

**Keywords:** electrolyte supplementation, interventions, nutritional management, pharmacological therapy, tubulopathies

## Abstract

Tubulopathies comprise a heterogeneous and still poorly defined group of inherited and acquired disorders in which tubular transport is disproportionately impaired, resulting in chronic electrolyte and acid–base disturbances with downstream complications such as nephrocalcinosis, nephrolithiasis, bone fragility and, in some entities, progressive chronic kidney disease. Over the past decade, high-cost targeted therapies have transformed outcomes for a subset of tubulopathy-related disorders with secondary tubular involvement (e.g. anti–fibroblast growth factor 23 therapy in X-linked hypophosphatemia), yet comparable innovations remain largely unavailable for primary tubular transport defects and access to these therapies is uneven across healthcare systems. In this narrative review, we synthesize current evidence and expert recommendations for pragmatic, phenotype-driven interventions that support daily care and may help prevent complications. Core management still relies on non-targeted measures, including individualized nutritional counselling, optimization of hydration and solute load, and tailored electrolyte and alkali supplementation. We also discuss the rational use of widely available drugs that can be repurposed in selected tubular phenotypes, such as thiazide and thiazide-like diuretics, potassium-sparing agents, azole antifungals in calcitriol-driven hypercalcaemic states and emerging data on sodium-glucose cotransporter 2 inhibitors. Finally, we emphasize supportive care components often overlooked in ‘drug-centred’ approaches, including structured patient education, adherence support and monitoring of extra-renal manifestations. Overall, this review argues for a more resource-conscious approach to tubulopathies: one that recognizes the lack of a consensual definition, prioritizes phenotype-based care bundles, and underscores the urgent need for larger prospective studies incorporating patient-reported outcomes and robust economic endpoints, particularly the out-of-pocket burden borne by patients and families.

## INTRODUCTION

Inherited and acquired tubulopathies represent a heterogeneous group of disorders in which renal tubular transport is disproportionately affected compared with glomerular function [1]. Yet, there is currently no consensual definition of what should be included under the umbrella term ‘tubulopathies’, and the existing literature does not provide a clear framework for this entity. Restricting the concept to diseases with a primary tubular transport defect [such as Gitelman or Bartter syndromes, or nephrogenic diabetes insipidus (NDI)] is probably too narrow, as several conditions with extratubular factors (for example X-linked hypophosphatemia) or enzymatic defects secondarily drive tubular dysfunction (for example primary hyperoxaluria). For some of these entities, it even remains debatable whether they should be regarded as true tubulopathies or as tubulopathy-related disorders.

Over the past decade, no disorder with a primary tubular transport defect has benefited from innovative, targeted therapies, whereas other diseases indirectly affecting the tubule (such as X-linked hypophosphatemia treated with burosumab [2] or primary hyperoxaluria treated with RNA interference therapies [3–6]) have seen their care transformed. These agents are, however, expensive, subject to stringent reimbursement rules, and not uniformly accessible across countries and healthcare systems [7].

In primary tubular transport defects, management instead relies on bundles of interventions that are, taken individually, relatively inexpensive: electrolyte and alkali supplements, vitamins, dietary and lifestyle measures, and drugs already available for other indications (for example diuretics used in hypertension), and therefore generally considered ‘cheap’ and accessible. Yet, these so-called ‘cheap’ interventions can generate a substantial financial and logistical burden for patients, as they are not always reimbursed [8]. To date, there are no dedicated medico-economic studies evaluating the impact and cost–utility of these interventions in tubulopathies. The rarity, clinical heterogeneity and multiplicity of underlying genes and phenotypes have also limited the feasibility of randomized controlled trials and robust comparative effectiveness studies, so that many treatment strategies still rely on physiological reasoning, small case series and expert opinion.

In this context, the aim of this narrative review is not to provide an exhaustive catalogue of all tubulopathies or all possible treatments, which would be neither feasible nor conceptually sound given the lack of a clear definition of the entity. Instead, we focus on a set of tubular disorders in which chronic electrolyte derangements and their correction are central to daily care: hypercalciuric tubulopathies, salt-wasting tubulopathies, tubulopathies with hypomagnesaemia, tubulopathies with hypophosphatemia, tubulopathies with water-balance impairment, tubulopathies associated with inherited hypertension, and tubulopathies with a significant risk of chronic kidney disease (CKD). As will be illustrated throughout this review, many individual diseases fall into several of these categories simultaneously. We will synthesize current knowledge on low-cost and resource-conscious therapeutic interventions, highlight the major evidence gaps and outline perspectives for future studies aiming to improve global care.

## WHY SHOULD WE CONSIDER ECONOMIC BURDEN IN TUBULOPATHIES?

Despite the chronic nature of tubulopathies, the absence of curative treatment, and the lifelong need for monitoring and therapy, there are, to our knowledge, no dedicated health-economic studies addressing their management. No published work systematically explores the economic burden of these conditions, including relatively ‘common’ tubulopathies such as Gitelman syndrome. A major aspect of this burden was highlighted in a recent study by the European Patient Advocacy Group (ePAG) of the Tubulopathies Working Group, based on a self-reported survey conducted across multiple European Rare Kidney Disease Registry (ERKNet) expert centres [8]. This survey revealed substantial costs for patients, with inconsistent reimbursement of electrolyte supplements and more limited financial coverage in lower-income settings, leading to a significant out-of-pocket burden for families. In children, the need for intravenous or gastrostomy electrolyte supplementation may further increase resource use. In many countries, electrolyte supplements are still not recognized as essential therapies or even as medications, as they do not contain a classical active pharmaceutical ingredient, which further hinders their formal inclusion in reimbursement schemes and creates marked disparities between healthcare systems.

**Figure 1:**
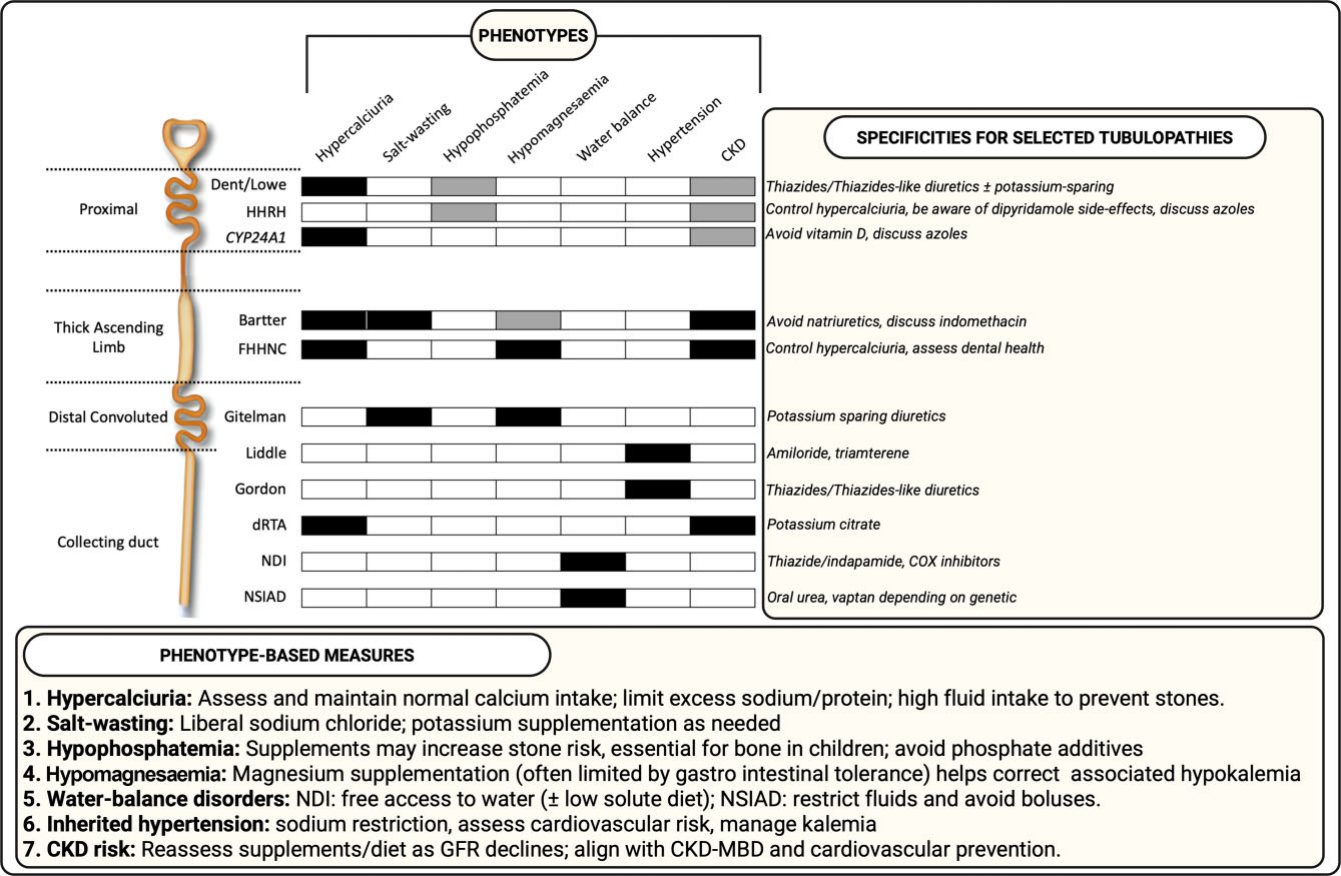
Phenotype-based matrix across selected tubulopathies. Schematic overview of a phenotype-driven approach to tubulopathies including a matrix positioning selected disorders across seven recurring clinical phenotypes (hypercalciuria, salt-wasting, hypophosphatemia, hypomagnesaemia, water-balance disorders, inherited hypertension and CKD risk). White indicates no typical involvement of the phenotype, black indicates a feature that is usually present or near-constant and grey indicates variable involvement depending on genotype and/or individual phenotype. This figure summarizes interventions based on the phenotype (lower panel) or specific tubulopathies (right panel). GFR, glomerular filtration rate. Created in BioRender.

Beyond the direct cost of long-term electrolyte supplementation, the overall economic impact of tubulopathies is likely to vary according to several factors: development of CKD or end-stage kidney disease, age at onset (childhood vs adulthood), quality-of-life impairment, and potential transmission to future generations. A first key distinction is between tubulopathies associated with CKD and possible progression to kidney failure (Fig. 1), and those in which CKD is absent or mild. In the former group, long-term costs include not only chronic supplementation and monitoring but also recurrent hospitalizations, and ultimately dialysis or transplantation. A second key distinction relates to the potential cost of kidney stones in hypercalciuric tubulopathies, including hospitalizations, surgical interventions and, in some cases, progression to kidney failure. In patients without CKD, the burden may be predominantly ‘functional’ in a substantial proportion of cases: fatigue, cramps and other consequences of chronic electrolyte losses can markedly impair quality of life, school attendance and work capacity, while the cumulative cost of medical care and the day-to-day professional and personal impact remains largely invisible in the absence of structured data. Large-scale information on sick leave, reduced working hours or disability is completely lacking. Existing expert recommendations for tubulopathies provide practical guidance on diagnosis, pharmacological and nutritional management, and follow-up schedules. Some explicitly acknowledge that socioeconomic difficulties, lack of reimbursement, adolescence, transition periods and work conditions may hamper adherence to supplements: ‘Physicians also should be attentive to other factors that could hamper adherence to the supplements, including socioeconomic difficulties, lack of reimbursement, adolescence, transition period, work conditions’ [9]. However, they do not incorporate explicit economic evaluation or stratification by resource level, and they do not address indirect costs such as travel to reference centres, caregiver time or lost productivity. This profound gap between clinical recommendations and economic data is one of the motivations for the present review and underscores the need for future studies specifically designed to capture both the therapeutic and economic burden of tubulopathies, in order to support more genuinely resource-conscious decisions for patients.

**Key messages: Why consider the economic burden in tubulopathies?**

- Tubulopathies require lifelong management but lack dedicated health-economic evaluation. Patients face substantial and often poorly reimbursed costs, particularly for electrolyte supplements, marked disparities between healthcare systems, and significant indirect burdens related to CKD progression, kidney stones and impaired quality of life.
- Addressing this gap is essential to support more resource-conscious and equitable care.

## HOW SHOULD WE DEFINE TUBULOPATHIES?

The term tubulopathy derives from tubulus (small tube) and pathos (disease) and refers to disorders in which dysfunction of the renal tubules is the predominant pathological feature. However, it remains unclear whether this category should be restricted to primary tubular defects (i.e. genetic alterations directly affecting tubular transport proteins) or whether it should also encompass conditions characterized by secondary or broader tubular dysfunction, such as disorders affecting specific tubular functions (e.g. X-linked hypophosphatemia), generalized proximal tubular dysfunction (Fanconi syndrome) or diseases in which tubular injury arises from enzymatic defects [e.g. primary hyperoxaluria or adenine phosphoribosyltransferase (APRT) deficiency].

A purely genetic or segment-based classification is of limited practical value for management, as therapeutic decisions are largely driven by the resulting disturbances in homeostasis rather than by the molecular defect itself. In clinical practice, both patient symptoms and treatment strategies are primarily determined by the dominant biochemical phenotype, namely, which electrolytes are chronically lost and whether kidney function is preserved or impaired. For the purposes of this review about interventions, we therefore organize tubular disorders into seven non-mutually exclusive phenotype-based categories: (1) hypercalciuria, often complicated by nephrocalcinosis, nephrolithiasis or osteoporosis; (2) salt-wasting tubulopathies; (3) renal hypophosphatemia; (4) renal hypomagnesaemia; (5) tubulopathies with water-balance defects; (6) tubulopathies with inherited hypertension; and (7) tubulopathies associated with CKD. The numbering of these categories (with the exception of category 7) does not imply any gradient of severity or prognosis.

In this framework, each individual tubulopathy may fall into one or several of these categories. For example, Dent disease, a proximal tubulopathy, combines marked hypercalciuria, renal phosphate wasting and progressive CKD (categories 1 + 3 + 7). Bartter syndromes, which involve a defect in the thick ascending limb, are characterized by combined calcium, magnesium and sodium losses and CKD (categories 1 + 2 + 4 + 7). Distal renal tubular acidosis associates hypercalciuria, nephrocalcinosis and a substantial risk of CKD, bridging categories 1 and 7. By contrast, Gitelman syndrome predominantly fits within the salt-wasting and hypomagnesaemia phenotype (categories 2 + 4).

In practice, most tubulopathies due to primary defect can be mapped to one or more of these seven categories, and this mapping is more informative for daily therapeutic management than a purely genetic classification. Figure 1 proposes a matrix in which each disease is positioned according to its phenotype, together with the main interventions usually required.

**Key messages: How should tubulopathies be defined?**

- Tubulopathies encompass a heterogeneous spectrum of disorders with predominant tubular dysfunction, which may range from primary transport defects to secondary or enzymatic conditions affecting tubular function. Because treatment is driven by disturbances in electrolyte and water homeostasis rather than by genotype alone, a phenotype-based classification, centred on dominant biochemical abnormalities, offers a clinically relevant framework to guide management.

## CAN DIETARY MANAGEMENT BE A COST-EFFECTIVE INTERVENTION IN TUBULOPATHIES?

Nutritional guidance is central to the management of tubulopathies, given the chronic disruption of electrolyte and mineral homeostasis. Dietary measures should be adjusted in the context of CKD, as progressive decline in glomerular filtration rate may mitigate urinary ion losses, and nutrition can influence CKD progression.

**Figure 2:**
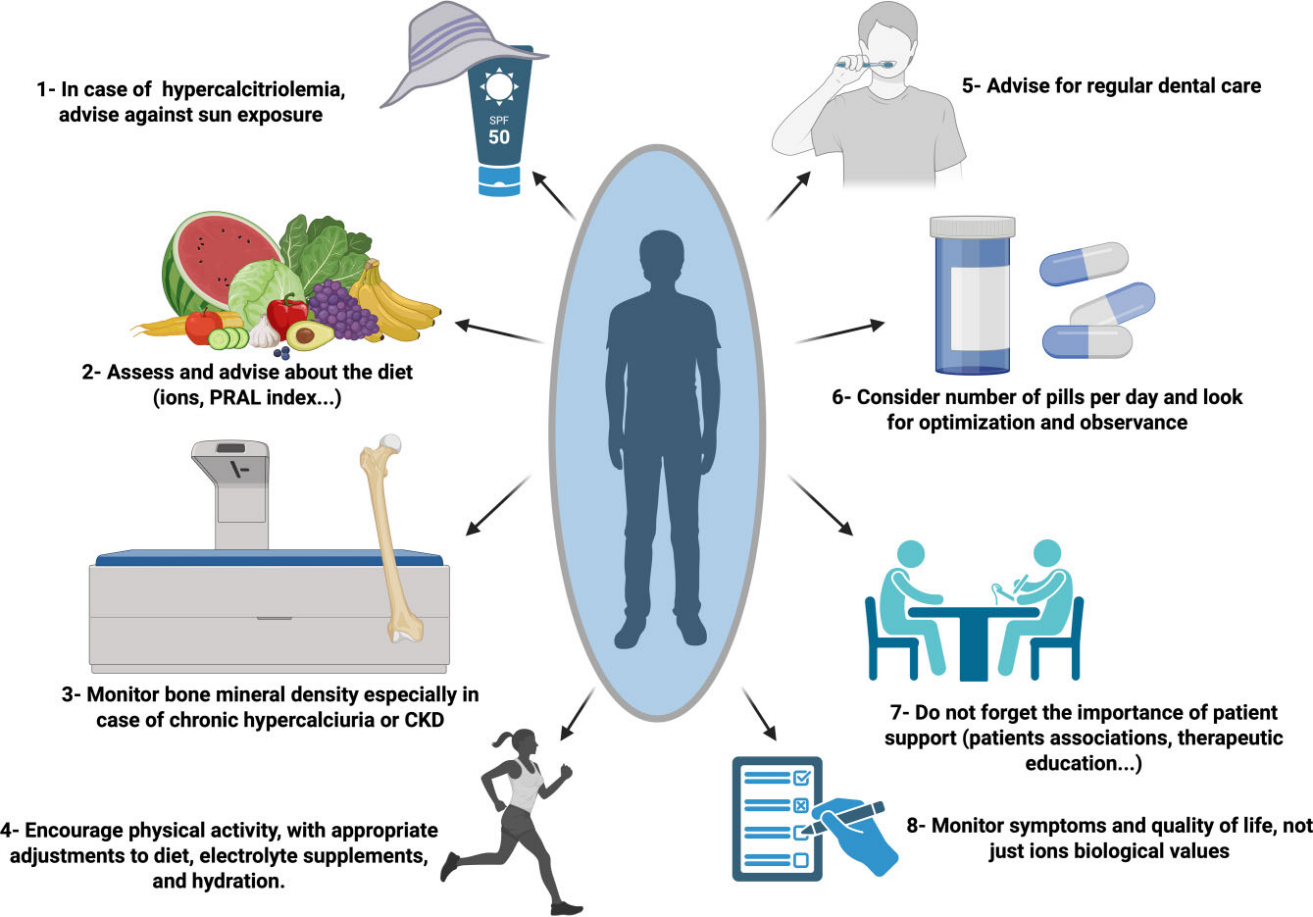
Useful non-pharmacologic interventions in tubulopathies. This figure highlights complementary, patient-centred interventions that optimize long-term management in tubulopathies. Monitoring symptoms and patient-reported outcomes, beyond laboratory values, is critical for holistic care in rare tubulopathies. Created in BioRender.

Patients should be educated on how to adjust their diet based on environmental factors such as heat, hydration status and physical activity (Fig. 2).

### Category 1: hypercalciuria

In hypercalciuric patients [e.g. Bartter syndrome, familial hypomagnesaemia with hypercalciuria and nephrocalcinosis (FHHNC), Dent/Lowe syndrome or distal renal tubular acidosis, see Fig. 1], maintaining a normal dietary protein intake is essential, as high protein consumption increases urinary calcium excretion through acid load and enhanced glomerular filtration [10]. Similarly, a high sodium intake is associated with increased calciuria [11]. By contrast, in patients with salt-wasting tubulopathies like Bartter syndrome, the benefits of increased sodium and chloride intake compensate for renal losses outweigh the associated risk of calciuria increase. It is needed in children to maintain growth, and in adults to help to maintain quality of life, which is frequently altered by chronic extracellular dehydration.

Calcium intake should not be restricted. A low-calcium diet increases intestinal oxalate absorption, thereby promoting calcium oxalate stone formation [12, 13]. Furthermore, in hypercalciuric patients, insufficient calcium intake may exacerbate bone demineralization. Conversely, excessive calcium intake can increase calciuria. Therefore, calcium intake should be adapted to the patient’s age, sex and hormonal status, and distributed throughout the day, for instance, by consuming two to three servings of dairy per day, ideally taken with meals to reduce oxalate absorption.

Increasing hydration is another strong recommendation, particularly in stone-forming tubulopathies. A fluid intake of at least 2.5–3 L per day is advised, preferably as plain water, while avoiding sugary beverages. Fluid intake recommendations should be individualized according to usual urinary output, the type of tubulopathy and the presence of extrarenal losses. Most patients can be advised to drink *ad libitum*. In patients with progressive nephrocalcinosis or recurrent kidney stones, fluid intake should be adjusted to the severity of the condition. Because monitoring total fluid intake can be challenging, given the contribution of various beverages and water from food, patients may instead monitor their urinary volume and colour as practical indicators. Tap water is a simple, cost-effective option and should be recommended whenever safe and appropriate, depending on geographic location.

### Category 2: salt-losing tubulopathies

Patients with salt-losing tubulopathies (e.g. Gitelman or Bartter syndromes, see Fig. 1) require adequate sodium intake. However, patient counselling can be complex in the context of high blood pressure, an uncommon feature in Gitelman syndrome prone to low blood pressure [14] despite insulin resistance [15].

While infants typically self-regulate their sodium intake according to physiological needs, adults may under- or over-consume salt due to cultural beliefs, dietary trends or inappropriate medical advice. No consensus currently exists regarding optimal sodium intake in Gitelman syndrome—it is suggested to offer *ad libitum* sodium intake [9]. A prospective clinical trial (NCT04995627) is ongoing to assess the effects of sodium chloride supplementation on biological markers and quality of life in this population. In paediatric patients, adequate sodium intake is especially important to support growth [9]. In contrast, sodium replacement in adults with CKD or hypertension must be carefully individualized. Sodium chloride should be recommended for patients with metabolic alkalosis, while sodium bicarbonate should be avoided, as it can worsen alkalosis and exacerbate hypokalemia.

Patients with Gitelman or Bartter syndrome may also benefit from foods rich in potassium and magnesium, though dietary sources are often insufficient, making supplementation necessary. Tables summarizing this high content food are available in the consensus paper on Gitelman management [9]. Consuming foods rich in sodium, chloride, potassium and magnesium before exercise can help prevent symptoms related to electrolyte depletion.

### Category 3: renal hypophosphatemia

In renal phosphate-wasting disorders, phosphate supplements from diet or medication do not correct the underlying tubular defect. The renal threshold for phosphate reabsorption remains unchanged, so supplementation increases the filtered phosphate load, which is then partly lost in the urine, sometimes substantially. This rise in phosphaturia may promote nephrolithiasis in patients with coexisting hypercalciuria. Thus, in patients with renal hypophosphatemia due to proximal tubular dysfunction, careful phenotypic assessment is required, including evaluation of kidney function as well as the presence [e.g. in Dent disease, Lowe syndrome, hereditary hypophosphatemic rickets with hypercalciuria (HHRH)] or absence (e.g. in cystinosis) of hypercalciuria.

Nutritional counselling is essential to support bone mineralization (and growth in children) and to alleviate symptoms potentially related to hypophosphatemia (e.g. cramps). Adequate phosphate intake from natural food sources (dairy products, meat, fish, eggs, nuts, whole grains) is desirable to complement pharmacological supplementation, whereas ultra-processed foods rich in phosphate additives should be discouraged [16]. As in hypercalciuric disorders, and in order to maintain bone homeostasis, calcium intake should be monitored and kept within age- and sex-specific recommendations.

### Category 4: renal hypomagnesaemia

In renal hypomagnesaemia, dietary measures alone are rarely sufficient to normalize serum magnesium levels, but they remain an important, low-cost adjunct to pharmacological supplementation. Patients should be encouraged to incorporate magnesium-rich foods into their daily diet, such as green leafy vegetables, legumes, nuts and seeds, whole grains, cocoa-based products and mineral waters with a high magnesium content. A table summarizing this high content food is available in the consensus paper on Gitelman management [9]. These measures may help to reduce the dose of pharmacological supplements needed, which are sometimes not reimbursed for the patients, and possibly improve gastrointestinal tolerance by spreading magnesium intake throughout the day.

### Category 5: water balance

In tubulopathies with major urine concentration defects, such as NDI, *ad libitum* access to fluid in all patients is essential to prevent from dehydration, hypernatraemia, constipation and growth failure in children [17]. The adequate level of hydration will be determined by the patients due to the absence in this disease of osmosensors alteration, making them capable of self-regulation. Across all ages, plain water should be the primary beverage; sugar-sweetened drinks, fruit juices and caffeinated beverages should be limited, as they add unnecessary calories, may worsen glycaemic control, increase diuresis and promote dental caries. Practical advice on distributing fluid and food intake throughout the day (including before and after physical activity, during hot weather or intercurrent illness) is crucial to avoid episodes of dehydration, particularly when patients must comply with fasting periods for medical procedures. Clinicians should regularly monitor clinical status and natraemia and be especially cautious in patients with neurological comorbidities, in infants, and in situations where self-regulation may be impaired (e.g. gastroenteritis). In infants and young children, nasogastric tube feeding or gastrostomy should be discussed when oral intake is insufficient to maintain adequate hydration and growth, particularly in the presence of vomiting [17].

Besides water intake, it is also important to avoid excessive salt and protein, as both increase the osmotic load that must be excreted by the kidney, thereby raising obligatory urine output and increasing the burden on water intake. Simplified formulae to guide paediatric prescriptions can be used [17] to achieve this balance without compromising growth through overly strict restriction. Families should be counselled to avoid very salty processed foods, fast food and high-protein diets, while favouring home-cooked meals with controlled salt content, balanced protein sources and sufficient energy density to support growth in young children.

In contrast, nephrogenic syndrome of inappropriate antidiuresis (NSIAD) is an X-linked disorder caused by activating mutations of the arginine vasopressin receptor 2 (AVPR2), resulting in inappropriately concentrated urine and chronic hyponatraemia [18, 19]. Dietary and behavioural measures also play a central role in management. Excessive fluid intake should be avoided, as even modest water loads may rapidly worsen hyponatraemia. Patients should be advised to distribute fluid intake evenly over time and to avoid sudden or large boluses of liquids. Whether increasing solute intake (salt and protein) can meaningfully improve the phenotype by raising urinary osmole excretion and thereby increasing free water clearance, remains to be established.

### Category 6: tubulopathies with inherited hypertension

A subset of tubulopathies presents with hypertension driven by inappropriate distal sodium chloride retention, typically with suppressed renin (and variably low aldosterone). This group includes Liddle syndrome (ENaC gain-of-function), Gordon syndrome/pseudohypoaldosteronism type II (PHAII) (WNK-pathway disorders with increased NCC activity) and apparent mineralocorticoid excess (AME) (11β-HSD2 deficiency with cortisol-mediated mineralocorticoid receptor activation). Dietetic care prioritizes sodium restriction (often a high-yield intervention) and avoidance of large, abrupt salt loads; potassium intake should be individualized based on baseline kalemia. It may be particularly relevant in PHAII, where hyperkalemia is typical; however, emerging data suggesting potential benefits of higher dietary potassium intake for blood pressure control and, in selected CKD populations, cardiovascular outcomes, mean that dietary advice in PHAII should be individualized and requires specific evaluation [20–23].

### Category 7: tubulopathies with CKD risk

When glomerular filtration declines in patients with tubulopathies, urinary losses of sodium, potassium, magnesium, calcium and phosphate tend to decrease, and some of the initially liberal strategies used to compensate tubular losses need to be progressively adjusted according to laboratory results. In parallel, diet should follow general CKD recommendations to limit cardiovascular risk, the main cause of morbidity and mortality in this population, by promoting a healthy weight and preventing or controlling obesity and diabetes. Details of the recommendations are available in KDOQI guidelines [24].

**Key messages: Can dietary management be a cost-effective intervention in tubulopathies?**

- Dietary management is essential in tubulopathies and, although rarely sufficient on its own.
  1. Hypercalciuria: normal calcium intake, limit excess sodium/protein, ensure high fluid intake to prevent stones.
  2. Salt-wasting: liberal, individualized sodium intake, dietary potassium/magnesium supportive but insufficient alone.
  3. Hypophosphatemia: avoid phosphate additives, assess stone/CKD risks.
  4. Hypomagnesaemia: magnesium-rich foods are useful adjuncts, rarely sufficient alone.
  5. Water-balance disorders: free access to water in NDI/restrict fluids and avoid boluses in NSIAD.
  6. Inherited hypertension: sodium restriction, individualized potassium intake.
  7. CKD risk: reassess dietary needs as GFR declines, follow CKD and cardiovascular prevention guidelines.

## IS ELECTROLYTE SUPPLEMENTATION TRULY A ‘CHEAP’ INTERVENTION?

Ion supplementation is generally required in tubulopathies with renal electrolyte losses (Fig. 1, categories 1, 2, 3, 4), as dietary intake alone is insufficient to compensate for ongoing urinary losses, and may be associated with high sugar content, potentially contributing to metabolic syndrome. Electrolyte needs may vary with seasonal temperature changes, physical activity, intercurrent conditions or the presence of gastrointestinal symptoms. Moreover, ion loss severity differs among patients, even within the same genetic variation (such as siblings with Gitelman syndrome) highlighting interindividual variability whose underlying pathophysiology remains poorly understood. Importantly, most electrolyte supplements are taken several times daily and over the long term, and are not consistently reimbursed across healthcare systems, making them a non-negligible and often underestimated out-of-pocket expense for patients [8].

### Category 1: hypercalciuria

In patients with hypercalciuria, dietary calcium intake should be systematically assessed, for instance using simple dietary questionnaires such as the Fardellone test [25]. Calcium intake should not be restricted, as insufficient intake may worsen bone demineralization and increase intestinal oxalate absorption, such as the risk of oxalate calcium stones [13]. In patients with low dietary calcium intake, and particularly in those at risk of osteopenia or osteoporosis, calcium supplementation should be considered. Calcium carbonate may be used at modest doses (250–800 mg/day), preferably taken with meals, with the aim of reaching age-appropriate recommended intake without excessive calcium loading or unnecessary increases in urinary calcium excretion.

### Category 2: salt-wasting tubulopathies

In salt-wasting tubulopathies, chronic urinary loss of sodium and chloride result in extracellular volume contraction, which triggers secondary hyperaldosteronism. This compensatory mechanism often leads to persistent hypokalemia and metabolic alkalosis. Therefore, sodium and chloride supplementation is a cornerstone of management: it helps restore extracellular volume, suppresses renin–angiotensin–aldosterone system activation and subsequently reduces renal potassium wasting. Sodium can be supplemented through sodium chloride preparations and/or increased dietary salt intake. The requirement for sodium and chloride varies depending on factors such as age, body size, dietary habits, physical activity, extrarenal losses (e.g. sweating, perspiration, gastrointestinal losses), and the severity of renal salt-wasting. Chloride repletion is also essential, as it contributes to the correction of metabolic alkalosis. Clinical goals include symptom relief (e.g. fatigue, dizziness, hypotension) and improvement in overall quality of life. In paediatric patients, adequate sodium/chloride supplementation is essential to support proper growth, which should be regularly monitored to guide and adjust dosing as needed.

Potassium supplementation is typically administered orally as potassium chloride or potassium citrate. The latter promotes alkalinization and should be avoided in patients with metabolic alkalosis. However, potassium citrate is an effective and inexpensive option for hypokalemic tubular acidosis. While potassium-rich diets may support overall intake, they are rarely sufficient to normalize serum potassium levels in hypokalemic patients. Potassium gluconate, although an option, should be used with caution due to its high glucose content, which may not be the best option for individuals with insulin resistance or significant dental concerns. However, it can be particularly beneficial around periods of physical activity, when both energy and potassium requirements increase. The required dose of potassium supplements should be tailored according to kidney function, especially in patients with Bartter syndrome, where CKD may alter potassium excretion over time. In patients with nephrocalcinosis, kidney stones and/or metabolic acidosis [such as distal renal tubular acidosis (dRTA)], potassium citrate may be preferred, as it also helps acid–base homeostasis and helps to reduce calcium crystallization in the urine. While ADV_7103_, a new combination of potassium citrate and potassium bicarbonate, is marketed for dRTA [26], its high cost limits accessibility. Alternatively, potassium citrate is available as generic tablets or effervescent powders to dilute in water, which are significantly less expensive. Gastrointestinal tolerance is variable depending on the patient and the dose, and symptoms typically appear shortly after starting citrate therapy. Patients should be advised to increase citrate dilution if gastrointestinal side effects occur (e.g. bloating, diarrhoea). However, in some cases, treatment discontinuation may be necessary, as these adverse effects can significantly impact quality of life.

### Category 3: renal hypophosphatemia

Although phosphate supplementation is often considered in tubulopathies characterized by renal phosphate wasting its use requires caution. In cases of renal hypophosphatemia, the phosphate reabsorption threshold in the proximal tubule is influenced by various factors, including circulating levels of fibroblast growth factor 23 (FGF23), parathyroid hormone or direct tubular dysfunction (Fanconi syndrome or specific alteration of phosphate reabsorption such as *SLC34A1* and *SLC34A3* variants). When tubular reabsorption is impaired, phosphate supplementation will increase urinary phosphate loss, thereby may exacerbate the risk of calcium phosphate stone formation. Excessive phosphate intake may also favour secondary hyperparathyroidism, vascular calcifications and nephrocalcinosis. However, data are scarce and contradictory between studies and depending on the physiopathology of each tubulopathy [27]. In paediatric patients, phosphate supplementation remains important to prevent rickets and osteomalacia [28]. Fractionated dosing throughout the day is recommended, to avoid a peak in phosphaturia and to help serum phosphate stabilization during the nychtemere. Therapies to reduce calciuria have to be considered as a first-line option in adults.

### Category 4: renal hypomagnesaemia

Hypokalemia and hypomagnesaemia are common and often coexisting abnormalities. Hypomagnesaemia contributes to renal potassium wasting by impairing Na^+^/K^+^-ATPase function and increasing renal outer medullary potassium (ROMK) channel activity, thereby aggravating hypokalemia [29]. Magnesium supplementation is therefore essential not only to restore magnesium levels but also to facilitate correction of hypokalemia. However, both potassium and magnesium supplements present challenges. Gastrointestinal side effects are frequent: magnesium salts commonly induce diarrhoea, while potassium chloride may cause gastric irritation and, in rare cases, peptic ulceration or bleeding [30]. Various magnesium salts are available; organic salts such as magnesium lactate or magnesium citrate offer better bioavailability compared with inorganic salts like magnesium oxide or magnesium sulfate [9, 31]. Slow-release preparations may improve tolerance and adherence. In expert centres, subcutaneous magnesium infusion or intravenous administration can be considered for severe, refractory hypomagnesaemia, though intravenous magnesium may be veinotoxic (due to high osmolarity) and should be used cautiously [9].

**Key messages: Is electrolyte supplementation truly a ‘cheap’ intervention?**

- Electrolyte supplements require multiple daily doses over years and are not consistently reimbursed, resulting in a substantial out-of-pocket burden for many patients.
  1. Hypercalciuria: assess and maintain normal calcium intake.
  2. Salt-wasting: sodium chloride is cornerstone, potassium as needed.
  3. Hypophosphatemia: phosphate supplements do not correct the defect and may increase stone risk, use cautiously, fractionated, essential for bone in children.
  4. Hypomagnesaemia: magnesium supplements help correct hypokalemia, tolerance limits adherence.

## ARE THERE LOW-COST THERAPIES APPLICABLE TO TUBULOPATHIES?

Pharmacological treatments do not always play a central role in the management of tubulopathies, where long-term electrolyte supplementation, nutritional measures and monitoring often dominate daily care. However, several drugs can provide meaningful benefit in tubulopathies (Fig. 1). There is no definition of what constitutes a ‘cheap’ treatment. Drug prices vary widely between countries and even within the same country over time. In addition, the effective cost to the patient is driven not only by the list price, but also by reimbursement rules and insurance coverage, which differ markedly between healthcare systems [8]. A medication that is considered low-cost and fully reimbursed in one setting may represent a substantial out-of-pocket burden in another. In practice, available drug price and reimbursement databases are fragmented and not directly comparable across countries. In this context, most of the interventions discussed in this review are not novel therapies specifically developed for tubulopathies, but rather older, widely available drugs originally designed for other indications (e.g. hypertension) that have subsequently found a practical role in tubulopathies.

### Category 1: hypercalciuria

#### Calciuria-reducing therapies (thiazides and potassium-sparing diuretics)

Thiazide diuretics are used in the management of idiopathic hypercalciuria and recurrent calcium nephrolithiasis [32]. By reducing urinary calcium excretion, they have been shown to lower the risk of stone recurrence. Current guidelines recommend their use in patients with recurrent stone formation associated with documented hypercalciuria [33, 34]. Thiazide diuretics have long been used in calcium stone disease because they reduce urinary calcium excretion and were assumed to lower recurrence risk. Their benefit has recently been questioned by the NOSTONE trial [35], which did not show a significant reduction in stone recurrence with hydrochlorothiazide versus placebo, although interpretation is limited by the inclusion of non-hypercalciuric patients, heterogeneous stone composition and high sodium intake. In contrast, an observational cohort by Hsi *et al*. [36] suggested that thiazide administration was associated with fewer symptomatic recurrences, supporting a potential benefit in carefully selected, hypercalciuric stone-formers with optimized sodium restriction.

There are few data specifically addressing the use of thiazides in hypercalciuric tubulopathies. It is important to distinguish hypercalciuric tubulopathies with salt wasting from those without, because salt-wasting phenotypes are prone to hypokalemia and already suffered from volume depletion, making thiazide diuretics (and other natriuretic agents) contraindicated in this setting. Thiazide diuretics, including ‘thiazide-like’ agents such as chlorthalidone, may nevertheless have a role in hypercalciuric tubulopathies without salt wasting, such as absorptive hypercalciuria due to variants in cytochrome P450 family 24 subfamily A member 1 (*CYP24A1*), *SLC34A1* or *SLC34A3*, or in Dent disease [37]. In this category, in order to enhance efficacy, thiazide therapy should be combined with dietary sodium restriction, as high sodium intake counteracts the calcium-sparing effects of thiazides. Co-prescription with potassium-sparing diuretics (such as amiloride, spironolactone, eplerenone), can help mitigate the risk of hypokalemia and increase the hypocalciuric effect [37]. Effective dosing is typically higher than that used for hypertension (e.g. hydrochlorothiazide at 50 mg/day and indapamide at 5 mg/day [38]). However, in patients with underlying tubulopathies or low baseline blood pressure, dose titration may be limited by hypotensive effects.

Importantly, thiazides have been associated with an increased risk of melanoma; therefore, patients should be informed, and those with high-risk skin phenotypes should undergo dermatologic follow-up [39].

#### Vitamin D

Vitamin D supplementation plays a supportive yet critical role in the management of hypercalciuric tubulopathies. Vitamin D supplementation is essential for maintaining skeletal health, particularly in individuals at risk of bone fragility. Vitamin D insufficiency is widespread in the general population, with prevalence estimates ranging from 20% to over 80%, depending on geographic region, sun exposure and dietary habits [40, 41]. Deficiency is especially prevalent in dark-skinned ethnic probants, as melanin inhibits cutaneous vitamin D synthesis. Vitamin D supplementation is especially important in patients with CKD or tubulopathies affecting mineral homeostasis. Patients should be advised to maintain sufficient calcium intake, as vitamin D without sufficient calcium can paradoxically increase bone resorption and worsen bone fragility [42, 43]. Calcifediol (25-hydroxyvitamin D) should not be used in patients, except in cases of hepatic insufficiency, as it bypasses a potential defect in hepatic vitamin D hydroxylation. When supplementation is necessary, using native vitamin D (cholecalciferol) allows the body to regulate its hydroxylation more physiologically, supporting a safer and more controlled correction of deficiency.

There is currently no standardized guideline for optimal vitamin D targets in tubulopathies. Nonetheless, maintaining serum 25-hydroxyvitamin D levels in the sufficient range (typically ≥30 ng/mL) is generally recommended, especially in individuals with bone fragility or at high risk for fracture.

Caution is warranted though in hypercalciuric tubulopathies, where vitamin D administration can transiently increase urinary calcium excretion, potentially exacerbating nephrolithiasis and nephrocalcinosis [43]. In case of active kidney stones, a daily low-dose regimen of cholecalciferol (800–2000 UI of cholecalciferol) may be preferred over high-dose intermittent supplementation (e.g. 50 000–100 000 IU weekly or monthly). This strategy minimizes peaks in active vitamin D levels and reduces the risk of sudden calciuric spikes. However, clinicians must also consider the risk of poor adherence with daily dosing, and treatment should be individualized. Vitamin D need depends on absorption (increased if given in a lipidic meal), so vitamin D status should be monitored to adjust dosage.

In disorders of impaired vitamin D degradation, such as *CYP24A1* deficiency, supplementation is avoided unless a profound deficiency is present. If treatment is deemed necessary [e.g. in a patient with osteoporosis and/or very low 25(OH) vitamin D levels], a cautious approach using low daily doses needs to be discussed, with strict biochemical monitoring, and avoiding period of sun exposure [44]. High-dose regimens should be avoided.

#### Azole antifungal agents

Azole antifungal agents, which include imidazoles (e.g. ketoconazole) and triazoles (e.g. fluconazole), act primarily by inhibiting lanosterol 14α-demethylase, a cytochrome P450 enzyme critical for ergosterol synthesis in fungi. Beyond their antifungal properties, these agents have been shown to inhibit other cytochrome P450-dependent enzymes, including those involved in vitamin D metabolism, particularly 1α-hydroxylase and 24-hydroxylase [45]. This pharmacological action has led to their off-label use in the treatment of hypercalcaemia associated with increased calcitriol production.

Ketoconazole has been used successfully to treat hypercalcaemia in various settings, including primary hyperparathyroidism, granulomatous diseases such as sarcoidosis, and *CYP24A1*-related disorders [46, 47]. Several case reports support its efficacy in reducing serum calcium, urinary calcium excretion, and 1,25-dihydroxyvitamin D levels [48, 49]. In these reports, ketoconazole was typically administered at a dose of 200 mg three times daily, with treatment durations ranging from 2 months to 2 years. While these cases did not report adverse effects, the authors emphasized the need for liver function monitoring due to known hepatotoxicity risks.

Fluconazole, a triazole with a more favourable safety profile and greater availability worldwide, has also been employed in this context. In a case report of *CYP24A1* deficiency, fluconazole administered at 50 mg/day led to sustained reductions in serum and urinary calcium without evidence of hepatotoxicity, even with long-term use [49]. Although fluconazole is a weaker inhibitor of 1α-hydroxylase than ketoconazole, its lower toxicity makes it a potentially valuable therapeutic alternative in selected patients.

These agents may be particularly relevant in the management of tubulopathies associated with dysregulated vitamin D metabolism, including pathogenic variants in *CYP24A1, SLC34A1* or *SLC34A3*. In *CYP24A1* mutations, defective 24-hydroxylase activity impairs calcitriol inactivation, resulting in hypercalcaemia and hypercalciuria. Similarly, renal phosphate wasting caused by mutations in *SLC34A1* or *SLC34A3* can stimulate 1α-hydroxylase activity, further enhancing calcitriol synthesis and aggravating calcium overload. In such settings and if studies confirm their efficacy [50], the off-label use of azole antifungals may potentially be able to offer a low-cost, mechanistically targeted therapeutic option when standard interventions (e.g. dietary restriction, hydration, thiazides) are insufficient.

#### SGLT2 inhibitors

Sodium-glucose cotransporter 2 (SGLT2) is expressed in the proximal convoluted tubule, where it plays a key role in glucose reabsorption. SGLT2 inhibitors have demonstrated significant renoprotective effects in CKD especially. Recent studies have suggested a potential role for SGLT2 inhibitors in kidney stone recurrence in kidney stone patients [51, 52], though data in the context of tubulopathies remain limited. In healthy volunteers, empagliflozin was associated with a 45% increase in urinary citrate excretion [53, 54], a key anti-lithogenic factor, acting as an inhibitor of calcium crystal nucleation, growth and aggregation in the urine. These findings suggest that SGLT2 inhibitors could influence risk factors for nephrolithiasis and merit further investigation in patients with tubulopathies with hypercalciuria.

### Category 2: salt-wasting tubulopathies

#### Potassium-sparing diuretics

Potassium-sparing diuretics and other agents modulating the renin–angiotensin–aldosterone system have been proposed to mitigate hypokalaemia and metabolic alkalosis in Gitelman and Bartter syndromes. In Bartter syndrome, however, the primary defect in the thick ascending limb causes marked renal sodium and chloride loss, and sodium reabsorption in the distal nephron is a crucial compensatory mechanism to maintain intravascular volume. Inhibiting this adaptation with potassium-sparing diuretics may exacerbate natriuresis and lead to severe hypovolaemia, particularly in infants, potentially contributing to life-threatening events, including sudden death; these drugs should therefore not be used in Bartter syndrome [55]. In Gitelman syndrome, by contrast, potassium-sparing agents and renin–angiotensin–aldosterone system blockers may be discussed in patients with profound hypokalaemia, especially when high-dose potassium supplementation causes significant gastrointestinal side effects, although evidence on their long-term safety and efficacy remains limited [9].

#### Cyclooxygenase inhibitors

Indomethacin is occasionally used in Bartter syndrome to reduce prostaglandin-mediated salt wasting (typically 1–4 mg/kg/day divided into three to four doses). However, its long-term use is discouraged due to gastrointestinal and renal adverse effects, and it is generally reserved for short-term management [55]. The use of ibuprofen and celecoxib has also been reported in Bartter syndrome.

### Category 3: renal hypophosphatemia

In renal phosphate-wasting disorders, pharmacological strategies aim either to reduce calciuria (and thereby the lithiasis/nephrocalcinosis risk, see category 1) or to directly modulate tubular phosphate handling. Dipyridamole has been shown to reduce renal phosphate losses and increase serum phosphate levels in patients with low tubular phosphate reabsorption thresholds [56]. Nevertheless, its widespread use is limited by cardiovascular side effects and the requirement for administration up to four times daily due to its short half-life. In renal phosphate wasting disorders, thiazide diuretics remain a first-line treatment to reduce calciuria (see Category 1), while fluconazole may represent an hypothetic second-line option, pending the results of recent trials [50, 57].

### Category 4: renal hypomagnaesemia

In hypomagnaesemic tubulopathies, such as Gitelman syndrome, familial hypomagnesaemia with hypercalciuria and nephrocalcinosis, and Ras-related GTPase D (RRAGD)-related disorders, pharmacological options remain limited. Amiloride, in addition to its role in improving serum potassium levels, may also help to increase serum magnesium concentrations in some patients, even if data are scare, and may depend on the cause of magnesium homeostasis alteration [58–60]. Its use must be weighed against the risk of worsening volume depletion in salt-wasting phenotypes and should be accompanied by careful monitoring of blood pressure, renal function and serum electrolytes.

SGLT2 inhibitors are currently explored for their ability to correct electrolyte imbalances such as hypomagnesaemia and hyponatraemia [61]. In a recent study, de Baaij and collaborators demonstrated improved magnesaemia levels in patients with RRAGD-related tubulopathy treated with SGLT2 inhibitors, without magnesaemia normalization [60]. Although evidence is still preliminary, these observations suggest that SGLT2 inhibition might contribute to partial correction of hypomagnesaemia in selected tubular disorders, in addition to its established renoprotective effects.

### Category 5: water balance

In NDI, pharmacological options are limited and daily management relies primarily on non-pharmacological measures, including unrestricted access to water and a low-solute diet. Thiazide diuretics, indapamide and cyclooxygenase (COX) inhibitors can reduce urine output, particularly in infancy and early childhood, by inducing mild volume contraction and enhancing proximal sodium and water reabsorption [17, 62]. Thus, close monitoring of fluid balance, weight and biochemistry is recommended at the start of treatment and patients should be given as much control as possible to self-determine their fluid intake. Their long-term efficacy is modest, and treatment requires close monitoring to avoid complications such as hyponatraemia. Overall, this relative lack of effective, durable pharmacological options underscores the central role of optimized, low-cost non-pharmacological interventions in this phenotype. When patients reach adulthood, or even earlier, it is recommended to discontinue COX inhibitors due to potential nephrotoxicity.

In NSIAD, the best-described low-cost option is oral urea, which increases osmotic diuresis and thereby enhances free-water clearance. Oral urea is neither an electrolyte supplement nor a targeted tubular drug (osmotic therapy). Long-term fluid regulation combined with low-dose urea has been reported as effective and well tolerated in both children and adults [19]. Some patients may also benefit from intermittent (non-daily) dosing, for instance during periods of anticipated increased fluid intake or early clinical signs of falling plasma sodium.

Vasopressin receptor antagonists (vaptans) show mutation-dependent effects in NSIAD: they were reported as ineffective in some cases, whereas they may represent a therapeutic option for selected variants (e.g. I130N-V2R) [63], underscoring the need for genotype-guided evaluation and careful monitoring.

Finally, SGLT2 inhibitors (especially empagliflozin) are being explored in other causes of hyponatraemia due to syndrome of inappropriate antidiuresis, where randomized trials reported a greater rise in plasma sodium when added to standard care [64]. This supports the concept that glucosuria-driven osmotic diuresis can increase water clearance. However, their role in NSIAD remains to be established.

### Category 6: tubulopathies with inherited hypertension

Pharmacological treatment targets the dominant transporter pathway: amiloride (or triamterene) is first-line in Liddle syndrome to directly inhibit ENaC [20]. This results in reduction of blood pressure and correction of hypokalemia and metabolic alkalosis. Thiazide/thiazide-like diuretics are typically effective in PHAII by inhibiting NCC and improving both blood pressure and hyperkalaemia [21]. In AME, strategies may include mineralocorticoid receptor antagonists and approaches that reduce cortisol-driven MR activation, alongside salt restriction [21]. Control of blood pressure is important to reduce the risk of cardiovascular complications.

### Category 7: tubulopathies with CKD risk

Tubulopathies with CKD risk may benefit from SGLT2 inhibitor therapy. Of note, off-label use of SGLT2 inhibitors has also been reported in Fanconi–Bickel syndrome (a rare GLUT2 deficiency impairing basolateral glucose transport, where prognostic of renal function remains unclear), where seven people from five families demonstrated proximal tubular function improvement under therapy [65]. More data are needed to fully assess the metabolic benefits of SGLT2 inhibitors in tubulopathy patients with CKD.

Vitamin D supplementation is another intervention particularly relevant to CKD-associated tubulopathies, to prevent mineral bone disorders.

**Key message: Are there available ‘low-cost’ therapies applicable to tubulopathies?**

1. Hypercalciuria: thiazides/thiazide-like ± potassium-sparing, azoles if calcitriol-driven.
2. Salt-wasting: avoid natriuretics in Bartter, consider potassium-sparing diuretics in Gitelman.
3. Hypophosphatemia: control hypercalciuria, be aware of dipyridamole side-effects.
4. Hypomagnesaemia: amiloride, SGLT2 inhibitors to be evaluated in this indication.
5. Water-balance: NDI: thiazide/indapamide, COX inhibitors. NSIAD: oral urea, discuss vaptan according to genetic variant.
6. Inherited hypertension: amiloride (Liddle) or thiazide (PHAII).
7. CKD risk: SGLT2 inhibitors when appropriate.

## CAN ACCESSIBLE NON-PHARMALOGICAL INTERVENTIONS IN TUBULOPATHIES PREVENT COSTLY COMPLICATIONS?

From sun protection to therapeutic education, several low-cost strategies can significantly improve outcomes in tubulopathies (Fig. 2). These interventions are not ‘cheap’ in an absolute sense, but they are widely implementable, and their value lies in preventing complications that are clinically and economically costly (e.g. hospitalizations, fractures, dental procedures, acute metabolic decompensations). However, to date, no dedicated medico-economic studies have quantified the cost/utility of these preventive strategies in tubulopathies, and their economic relevance is therefore inferred rather than evidence-based.

### All categories

#### Empowering patients with educational tools

Patient education may be a critical, cost-effective intervention in tubulopathies, offering measurable benefits across adherence, knowledge and self-management. In CKD, low health literacy affects up to 25% of individuals and is correlated with increased morbidity and mortality [66].

Studies of structured therapeutic education programs, including ‘teach-back’ methods, have shown improvements in patient knowledge, self-efficacy and adherence, although direct effects on renal outcomes require further investigation [67].

Engagement with patient advocacy groups enables peer support, shared experiences and empowerment, which can reinforce adherence and disease comprehension.

Collectively, these interventions enhance patient engagement, empower self-management, and potentially reduce complications and progression in tubulopathies as in CKD broadly. In practice, education is particularly impactful in tubulopathies because daily care often depends on multiple small decisions (hydration, salt intake, timing of supplements), where adherence and anticipatory behaviours directly influence outcomes.

#### Physical activity

Exercise is beneficial for physical and mental health. Especially, in Gitelman syndrome, exercise can alleviate fatigue and muscle weakness, and reduce insulin resistance [15]. However, exercise must be accompanied by proper hydration and adequate intake of sodium, potassium or magnesium (depending on the chronic ion losses), particularly before physical activity to prevent exacerbation of electrolyte imbalances. This is especially important during hot weather or prolonged physical exertion, where fluid and salt losses are increased. Structured education on peri-exercise electrolyte intake can be provided at low cost.

### Category 1: hypercalciuria

#### Sun avoidance in case of *CYP24A1* variants

Inactivating variants of the *CYP24A1* gene, which encodes the 24-hydroxylase enzyme responsible for degrading active vitamin D (1,25-dihydroxyvitamin D), lead to elevated circulating calcitriol levels, hypercalcaemia and nephrocalcinosis. In these patients, cutaneous vitamin D synthesis via ultraviolet exposure exacerbates the biochemical phenotype [68]. Consequently, non-pharmacological photoprotection (including avoidance of peak sunlight hours, use of high-SPF sunscreen and protective clothing) is recommended as a first-line intervention. This strategy aims to reduce endogenous vitamin D production and mitigate calcium overload. It may help prevent the costs associated with a potential episode of hypercalcaemia.

#### Bone health monitoring

Patients with hypercalcitriolemic conditions, such as *CYP24A1* mutations, may be susceptible to reduced bone mineral density and osteoporosis [68].

Dual-energy X-ray absorptiometry is a cost-effective, non-invasive tool that enables the assessment of bone mineral density and fracture risk in patients with tubulopathies. When combined with regular monitoring of bone turnover markers, DXA contributes to a comprehensive evaluation of skeletal health and are part of guidelines in tubulopathies management, especially in case of hypercalciuria or other metabolic pathogeny at risk for bone demineralization, such as acidosis in dRTA [69].

In addition to pharmacological interventions, modifiable risk factors for bone fragility should be addressed. Weight-bearing physical activity, smoking cessation and adequate nutritional intake are key components of bone health maintenance and should be integrated into the multidisciplinary care of patients with tubulopathies.

#### Dental care

Dental abnormalities (e.g. enamel hypoplasia and dentin defects) have been reported in several tubulopathies with hypercalciuria, mainly as case reports or small series (including dRTA, FHHNC due to *CLDN16/CLDN19* variants, Dent disease, Bartter syndrome) [70–73]. In the absence of large cohorts, it remains unclear to what extent these manifestations reflect chronic ionic disturbances versus a direct effect of the causal variant on dental tissues. Some disorders support a gene-specific contribution (see category 4: hypomagnesaemia).

Regular dental surveillance is therefore essential, including preventive strategies (e.g. fluoride application, sealants) and early treatment of caries and periapical infections. Early involvement of paediatric dental teams, in close collaboration with nephrologists and geneticists, is recommended for children with genetically confirmed tubulopathies. Importantly, dental care should be recognized as an integral part of the management of these patients, and efforts should be made to ensure that dental health expenses are included in care plans to facilitate adequate reimbursement and access to preventive dental services.

### Category 3: renal hypophosphatemia

#### Bone health monitoring

Tubulopathies associate with hypophosphatemia also results in bone demineralization and rickets (if it occurs in childhood before bone maturation), or osteomalacia in adults. In addition to these systemic factors, there is ongoing debate about whether certain bone phenotypes might also be directly related to the underlying genetic defect—particularly in disorders involving *SLC34A1* or *SLC34A3*, which encode renal phosphate transporters [74]. Osteomalacia and osteoporosis are characterized by low bone mineral density, but management is different. These assessments are essential for guiding individualized calcium and vitamin D supplementation strategies. In renal hypophosphatemia, bone monitoring should explicitly address growth (in children), fracture risk and pain/function (in adults), as these outcomes drive disability and healthcare use.

#### Dental care

In X-linked hypophosphatemia, chronic phosphate wasting leads to defective dentin mineralization and is frequently complicated by spontaneous endodontic infections and abscesses [75]. This phenotype supports proactive dental surveillance, as infections may occur in the absence of obvious caries and may require urgent dental interventions.

### Category 4: renal hypomagnesaemia

#### Dental care

In familial hypomagnesaemia with hypercalciuria and nephrocalcinosis, *CLDN16* and *CLDN19* are expressed in dental tissues and are associated with enamel (and, for *CLDN19*, ocular) abnormalities [76–78]. In these syndromic tubulopathies, consultation in an expert centre may have strong benefit for adequate dental care.

### Category 7: tubulopathies with CKD risk

When tubulopathies are complicated by CKD, prevention of ‘costly complications’ becomes multifactorial, as CKD introduces additional drivers of morbidity beyond the tubular phenotype. Bone health risk is no longer only explained by the underlying tubular defect, but progressively integrates CKD mineral and bone disorder mechanisms (secondary hyperparathyroidism, altered vitamin D metabolism, phosphate retention). Monitoring strategies especially aim to prevent fractures and cardiovascular events, all of which carrying major personal and health-system costs.

**Key messages: Can accessible non-pharmalogical interventions in tubulopathies prevent costly complications?**

- Education, physical activity, sun avoidance in *CYP24A1* variants, dental and bone surveillance, may help to reduce complications that are clinically and economically costly. Further studies to assess their medico-economic effect are needed.

## CAN LOW-COST COMPOUNDS BE HARMFUL IN TUBULOPATHIES?

### Avoid vitamin C supplements in hypercalciuric patients

Unlike other vitamins that may play a supportive role in the management of tubulopathies, vitamin C (ascorbic acid) supplementation should be approached with great caution (or avoided entirely) in patients at risk of kidney stone formation, particularly those with hyperoxaluria or recurrent calcium oxalate nephrolithiasis. Ascorbic acid is a metabolic precursor of oxalate, and a portion of ingested vitamin C is converted to oxalate via hepatic metabolism. Several studies have demonstrated that high-dose vitamin C supplementation leads to increased urinary oxalate excretion, thereby enhancing the risk of calcium oxalate crystallization and kidney stone development, particularly in men [79–81].

In contrast, dietary vitamin C obtained from fruits and vegetables is not considered problematic, and are even recommended. Indeed, vitamin C doses are significantly lower than those found in supplements, and such foods are rich in citrate, which has a protective effect against stone formation by inhibiting crystallization.

### Be careful with loop diuretics in hypercalciuric or salt-losing tubulopathies!

Loop diuretics should be avoided in the management of tubulopathies, especially those associated with hypercalciuria (e.g. Bartter syndrome, FHHNC due to *CLDN16/CLDN19* variants or *CYP24A1* inactivation). Loop diuretics inhibit sodium and chloride reabsorption in the thick ascending limb of the loop of Henle, which also impairs calcium reabsorption, thereby increasing urinary calcium excretion. This exacerbates pre-existing hypercalciuria and raises the risk of nephrolithiasis and nephrocalcinosis. Their use should be restricted to acute situations involving volume overload (e.g. pulmonary oedema or hypervolemia in advanced CKD).

### Caution with supplements and nephrotoxic products in tubulopathies

An increasing number of patients are self-administering dietary supplements, including over-the-counter vitamin D formulations, often derived from naturopathic or non-standardized sources. Unregulated vitamin D supplements may lead to inadvertent overdosing and hypercalcaemia, especially in infants [82, 83]. This is particularly concerning in patients with hypersensitivity to vitamin D, such as those with *CYP24A1* mutations, in whom even small amounts of vitamin D can cause hypercalcaemia and promote nephrocalcinosis. These individuals must avoid non-prescribed vitamin D products entirely.

Patients with tubulopathies should also be counseled to avoid potentially nephrotoxic substances beyond oral supplements. For instance, recent reports have identified severe kidney injury associated with the use of glyoxylic acid–containing hair-straightening products [84]. These products can cause tubular damage even in individuals without pre-existing renal disease and are particularly hazardous in patients with compromised tubular function. As a rule, patients with inherited tubulopathies should be advised against self-medication and exposure to unregulated or potentially nephrotoxic substances to preserve renal function over time.

## DISCUSSION

Tubulopathies represent a heterogeneous and still poorly defined group of disorders. In this review, rather than aiming for an exhaustive catalogue of diseases, we focused on a limited number of recurring tubular phenotypes that are directly relevant to day-to-day patient care.

Within this framework, dietetic measures should be considered as a first-line intervention in most patients. Nutritional and hydration measures can partially (however rarely fully) correct chronic electrolyte and acid–base disturbances and support bone homeostasis (including growth in children), while remaining relatively low-cost and accessible. Dietetic measures need to be individualized according to the tubular phenotype, age of patient, comorbidities and to the presence or absence of CKD.

Because several tubulopathies are characterized by chronic renal ion losses, non-active pharmacologic supplements (electrolyte and alkali preparations) are essential components of care. Yet, these ‘cheap’ therapies remain surprisingly under-evaluated. Robust data on long-term clinical outcomes, quality of life, adherence and, above all, health-economic impact are scarce or absent. Most recommendations still rely on physiological reasoning, small case series and expert consensus, rather than on prospective comparative studies. Developing pragmatic registries and multicentre trials that incorporate patient-reported outcomes and economic endpoints should therefore be a major priority for the field.

The unit price of these supplements is often modest, but the combination of high daily doses, multiple intakes per day and lifelong treatment turns them into a substantial cumulative financial burden for patients and families [8]. Major disparities exist between countries in terms of pricing and/or reimbursement, and available data do not allow a comparative, multi-country analysis of these costs. This narrative review was not designed to perform a formal medico-economic evaluation and should not be interpreted as such; rather, the difficulties we encountered in accessing and comparing cost data strongly argue for a dedicated, collaborative effort to document the economic burden of tubulopathies in a standardized way.

In parallel, high-cost targeted therapies have transformed the outlook for a minority of tubulopathy-related disorders, such as anti-FGF23 antibodies in X-linked hypophosphatemia [85]. However, their availability is uneven, and they do not obviate the need for optimized, low-cost care bundles, especially in healthcare systems with constrained resources. Looking forward, newer pharmacological approaches, including SGLT2 inhibitors, may further broaden the therapeutic landscape, but they also need to be evaluated within this resource-conscious perspective.

Regarding extra renal phenotypes, we propose for patients with tubulopathies to structure follow-up around the dominant risks (stones/nephrocalcinosis, metabolic decompensations, extrarenal features and/or CKD progression). Beyond kidney outcomes, we emphasize that systematic attention to extra-renal complications is part of high-quality care, with bone health monitoring (to detect and differentiate osteomalacia versus osteoporosis) and risk- and syndrome-informed dental surveillance (particularly in disorders with established dental involvement), ideally coordinated through expert centres. While the prevalence and cost-effectiveness of these strategies are not yet supported by dedicated medico-economic studies, their rationale is to prevent clinically and economically costly complications and to improve long-term quality of life.

## CONCLUSION

Most patients with tubulopathies still depend on pragmatic, low-cost measures to control electrolyte disturbances, limit complications in the absence of targeted therapies. Nutritional strategies, generic supplements, ‘low-cost’ drugs, patient education and lifestyle adaptations therefore remain the backbone of care and must be tailored to the individual tubular phenotype and monitored over time.

Addressing current evidence gaps in the definition and treatments in tubulopathies will require coordinated efforts from reference centres, patient organizations and health economists to improve global care while limiting the financial and practical burden for patients and families.

## Data Availability

No new data were generated or analysed in support of this research.
